# New Perspectives in Phenomenological Psychopathology: Its Use in Psychiatric Treatment

**DOI:** 10.3389/fpsyt.2018.00466

**Published:** 2018-09-28

**Authors:** Guilherme Messas, Melissa Tamelini, Milena Mancini, Giovanni Stanghellini

**Affiliations:** ^1^Department of Mental Health, Santa Casa de Sao Paulo School of Medical Sciences, São Paulo, Brazil; ^2^Institute of Psychiatry- Hospital das Clínicas de São Paulo, São Paulo, Brazil; ^3^Department of Psychological Sciences Humanities and Territory, University of Chieti, Chieti, Italy; ^4^Diego Portales University, Santiago, Chile

**Keywords:** phenomenological psychopathology, psychiatric care, phenomenological approaches, psychotherapy, person-centered approach, life-world, dialectics, anthropological proportion

## Abstract

Phenomenological psychopathology is a body of scientific knowledge on which the clinical practice of psychiatry is based since the first decades of the twentieth century, a method to assess the patient's abnormal experiences from their own perspective, and more importantly, a science responsible for delimiting the object of psychiatry. Recently, the frontiers of phenomenological psychopathology have expanded to the productive development of therapeutic strategies that target the whole of existence in their actions. In this article, we present an overview of the current state of this discipline, summing up some of its key concepts, and highlighting its importance to clinical psychiatry today. Phenomenological psychopathology understands mental disorders as modifications of the main dimensions of the life-world: lived time, lived space, lived body, intersubjectivity, and selfhood. Psychopathological symptoms are the expression of a dialectical modification of the proportions of certain domains of the life-world or of the lived experience. The far-reaching relevance of the concepts of proportion and dialectics for the clinical agenda is explored. The article presents two contemporary models for clinical practice based on phenomenological psychopathology: Dialectical-proportional oriented approach and Person-centered dialectic approach (P.H.D. method). The main characteristics of these approaches are considered, as well as the new perspectives they bring to the challenges of psychiatric care in the twentieth-first century.

## Introduction

The association between phenomenology and psychopathology was first postulated by Karl Jaspers, in his seminal work, “General Psychopathology” (1913/1997). Since “General Psychopathology,” psychopathology has been understood as a body of scientific knowledge on which the clinical practice of psychiatry is based, a method to assess the patient's abnormal experiences from their own perspective, and more importantly, a science responsible for delimiting the object of psychiatry. Gradually, phenomenological psychopathology started to be understood not just as a description of the subjective experiences of patients suffering mental disorders, but as a search for their conditions of possibilities (1)—the structures of subjectivity that underpin the experience of reality, which, when modified, determine psychopathological life-worlds. Furthermore, phenomenological psychopathology in recent times has become the background knowledge from which “know-how” methods for treatment have developed. In this article, we present an overview of the current state of this discipline, summing up some of its key concepts, and highlighting its importance to clinical psychiatry today.

## Main dimensions of the life-world

Phenomenological psychopathology assesses the life-worlds of mental disorders. The life-world is the world each of us live by, the original, obvious, and unquestioned foundation of everyday acting and thinking. Next to the common-sense world we all more or less share, there are several frameworks of experience, as for example, fantasy worlds, dream worlds, and psychopathological worlds. In the latter, psychopathological symptoms are the expression of a modification of the framework within which they are generated. In each symptom the change in the framework of experience becomes manifested. The experience of time, space, body, Self, and others are the basic dimensions of the life-world within which each single symptom is situated (2, 3).

### Lived time

Lived time must be distinguished from the time of the clock (“objective” time). Lived time is the way we subjectivey experience time rather than the objective time of the clock (4). Every experience receives its specific significance and value from its temporal profile.

### Lived space

Lived space is the way people live space, that is, the totality of the space that a person prereflexively “lives” and “experiences.” This space is based on the relationship of the person to her world as a situated and embodied entity.

### Lived body

The term “lived body” is used to designate the body that is lived by us and distinguish it from the physical body. It is the body experienced from within, the body in the first-person perspective (5). The lived body is the center of three main dimensions of experience (6): (a) self-experience and especially the most primitive form of self-awareness; (b) object-experience and meaning-bestowing; (c) the experience of other people.

### Intersubjectivity

Intersubjectivity is a key factor for reality constitution. Pragmatically, it is the capacity to grasp the meaning of the other persons' behavior and expression (7). The majority of everyday relations are grounded on pre-reflexive encounters with other persons. The others' mental states, including emotions, beliefs, and desires are directly expressed in their actions and are typically grasped as meaningful in an emergent, pragmatic context. We are attuned with each other through a direct, prethematic contact with the expressive behavior of the others.

### Selfhood

The notion of “Self” comprises at least two different dimensions: the pre-reflexive Self and the reflexive Self (8). By “pre-reflexive Self” we mean the most primitive form of self-consciousness. This primitive experience of oneself, rooted in the lived body, does not arise in reflection and is not inferentiallly or criterially given. It is neither conceptual nor linguistic, but a primordial contact or acquaintance with oneself. Next to this dimension of self-consciousness there is an experience of one' s own Self that implies the possession of a concept of oneself. This is the Self as narrative identity—the Self that tells stories about itself that exists in those stories and conceives its identity in terms of those stories.

## The two psychopathological groups: the life-world of structural disorders and anthropological disorders

Mental disorders can be divided into two major groups, in consistency with the depth of the alteration of the structure of the life-worlds: structural and anthropological disorders (9). Structural disorders correspond to life-world disturbances in the strict sense, in which the very constitution of reality and the overall ontological framework within which the patient's existence takes place is at stake (10). The prototypical model of structural disorders is schizophrenia. In anthropological disorders, such as non-psychotic ones, e.g., phobias and neurotic obsessions, the overall constitution of reality is not compromised, but the anomalies can be seen as dialectical modifications of the proportions of certain domains of the lived experience. The far-reaching relevance of the concepts of proportion and dialectics for all mental disorders and for the clinical agenda is explored in the following sections.

## Models for clinical practice based on phenomenological psychopathology

### Dialectical-proportional oriented approach

Dialectics is a notion that expresses the basic tensions and oppositions that permeate human life. It is the basis for understanding the immanent mobility of existence, the “*source* of constant movement [of existence]” [(11), p. 341]. The introduction of the notion of dialectical proportion into phenomenological psychopathology (12) is indicative of the considerable expansion of their heuristic and technical importance in the discipline at the present time. We will now examine a few of the reasons for this breadth of scope.

The notion of dialectical proportion:

- favors the observation of the complexities inherent to each mode of pathological experience, both structural and anthropological. In structural disorders, it allows psychopathological understanding to be extended to the most complex of clinical situations, in which identifying the person's relationship with her basic abnormal experience is what matters (13). For instance, schizophrenia can be understood not just from its core elements of delusion, but from the dialectical relationship between the loss of the constitution of reality and its maintenance (14).
- allows the themes of psychic movement and transformation to be introduced to the field of psychopathology (15, 16), offering diagnostic instruments that expand phenomenologically the observation of clinical course beyond a strictly biomedical sense (17).
- enables a conception of phenomenology-based care attuned with the latest needs of psychiatric practice. According to the current-day perceptions of clinical practice, treatment essentially consists of recovering from mental disorders (18). The centrality of the goals of treatment in the notion of recovery is generally in line with the idea of mental disorders having unique features, for which the conceptions of somatic medicine fall short. As such, the most reliable criteria for evaluating the development of a mental disorder have to do with the subject and his/her contexts, not external factors expressed in terms of signs or symptoms. For this reason, it is fundamentally important for the categorized mental disorders to reflect the dialectical aspect of the criteria. And, even more importantly, for these categories to allow a scientific view of the existential movements by which the person is renewed in the process of recovery. As such, offering a framework of psychopathological categories usable in clinical practice means offering categories that identify the movements of psychological transformation and evolution (15) through which recovery can be attained. Clinical practice depends strongly on this conceptual work, not only for setting the right therapeutic course, but also for appraising clinical developments.

There are two main forms of dialectical apprehension, which contributes distinctly to clinical practice.

- Dialectic of ambiguity
- Dialectic of anthropological proportions

#### Dialectic of ambiguity

This perspective picks up on the ambiguity of human experience (19). The ambiguity inherent to the human being reveals how all experience presents two simultaneous faces. As such, a “melancholic type” of personality (20) curbs the potential for personal expansion as it is too strongly linked to normality and duty in the performance of its social role; meanwhile, for the very same characteristics, a person with this personality structure is seen as someone to be trusted and respected by her family and community. Knowing this inherent ambiguity in existence is key to developing a therapeutic strategy for two reasons. First, understanding the ambiguities inherent to each pathological condition orientates therapeutic strategies, guiding more accurately the clinician's intervention and supporting her choice of certain procedures or conducts to the detriment of others. For instance, a distinctive characteristic of the “melancholic type” is intolerance of ambiguity (21, 22). Such people have almost insurmountable difficulty when they experience situations where any emotional indetermination is present. Faced with such circumstances, they tend toward existential restriction, retreating far from the world and themselves (20) and ultimately descending into a full-blown melancholic state. The clinician, aware of this limitation, should therefore: (i) find out what dilemmas the patient is unable to face; (ii) process and interpret their existential situation in terms that do not imply great existential risks; (iii) understand the extent and importance of these patients' existentially conservative values; (iv) stress the positive features of their potentialities, avoiding increasing the ambiguity in which the pathological state is immersed; (v) guide the pharmacological strategies along the same lines, following the same principles; (vi) select a method of treatment that leads to a resumption of the previous existential plans, as far as possible.

#### Dialectic of anthropological proportions

The dialectic of proportions is interested in a kind of microscope knowledge of *indeterminate* and *dynamic* elements. Elements that are *indeterminate* are so because they seek out something that is not yet known to the clinician or the patient—i.e., the tendency to have some kind of experience, which, emerging on the horizon of the patient's consciousness in the course of treatment, may influence its progress. It is a psychopathology of anticipation (23) or, to put it differently, a psychopathology of the middle ground between “what no longer is” and “what is yet to be.” The clinician seeks to incorporate the meaning of this indeterminism to the totality of the patient's existence to better conduct the case. An example of the importance of this indeterminism is the high risk of relapse in patients with substance addiction, even after having managed to abstain, when they are faced with a new challenge. The new challenge puts new demands on the patient in recovery—ones to which his/her consciousness may not yet be accustomed—because their lived time has been restricted to the present for so long (24, 25), making them incapable of opening up to the future. The demands of the future may, then, destabilize their existence, increasing their tension, and ultimately making them more vulnerable to relapse, which would constitute a return to the previous state of complete fusion with the world in the present (26). Such knowledge is extremely valuable for a clinician, because it prepares him/her to observe and monitor the patient closely, even when they are not using any substance, at these moments of anticipation of an experience and, when necessary, to take measures to protect them behaviorally. Thus, understanding the risk of relapsing extrapolates a merely behavioral perspective, expressed in terms of the capacity to resist the urge to use the substance, and takes on a broad existential meaning that is more in line with the profound characteristics of existence.

The *dynamic* aspect in the analysis of anthropological proportions picks up on tensions that are fundamental to existence: the tension between *permanence* and *becoming* and the opposition between *individuality* and *generality*. Throughout our lives, we are and we cease to be at one and the same time. Although we recognize our experiences and our memories as belonging to us, we know we are no longer the same as we once were, for instance, in our childhood. Phenomenological psychopathology examines the different ways existence transits between permanence and transformation (27), offering instruments for clinicians to lead each case through the characteristics and possibilities of each human type and each disorder. Clinical conduct based on examining the forms of the permanence-transformation dialectic focuses on allowing each existence to open up to the future wide enough and long enough for the personal identity to modify and become plural, while respecting the limits of each temperament and each individual.

Meanwhile, when it comes to the tension between individuality and generality, the quest for individualized treatment as part of person-centered care calls for the clinician to assimilate phenomenological observation categories that focus on the dialectics between the mental disorder and the individual meaning they take on for each person (24).

### The person-centered dialectic approach and the P.H.D. method

In this section we describe the overall framework into which the clinical approach based on dialectical proportions is applied. The aim of such a therapy is re-establish the dialectic between selfhood and otherness that will allow the suffering person to recover a sense of identity. The main principles of this approach can be summed up as follows (3):

- it supports the patient's unfolding his personal experience;
- it helps him to identify a core-meaning in his experiences around which his narrative can become meaningful;
- it incites him to make explicit his personal horizon of meaning, values and beliefs, within which her narrative is set;
- it also incites the clinician to make explicit to the patient her own assumptions, personal experiences, beliefs (at least, that part that is relevant for therapeutic purpose) on which her understanding of the patient's narrative is based;
- it promotes a reciprocal exchange of perspectives between the clinician and her patient;
- this “reciprocity of perspectives” is aimed to co-construct of a new meaningful narrative which includes and, if possible, integrates contributions from both the patient's and the clinician's perspectives;
- the clinician supports the patient to tolerate potential conflicts of values and beliefs and facilitates coexistence in case it is not possible to establish consensus.

The practice of care that derives from this is based on the integration of three basic dispositives, synthesized in the acronym PHD (28, 29):

*Phenomenological unfolding* (P): The explication of the patient's field of experience. This is done through a dialogue that opens up and lays bare the pleats of the patient's experiences and actions. Unfolding enriches understanding through recovering the implicit (not necessarily rejected), automatic (not censored), forgotten (not forbidden) sources that make phenomena appear as they appear to the patient, his drives, emotions, and habitus—the three emblematic components of the obscure and dissociated spontaneity that make up the involuntary dimension in human existence.
*Hermeneutic analysis (H): The explication* of the person's position-taking toward her experience. Since psychopathological symptoms, according to clinical hermeneutics, are the outcomes of an active interplay between the person and her basic anomalous, disturbing and dysfunctional experiences, attention is paid to the active role that the person has in taking a position and interacting with them. Rescuing from the implicit the active role that the patient has in shaping his symptoms is the *via regia* to help the patient to recalibrate his miscarried position-taking and, finally, to recover his sense of responsibility and agency.
*Dynamic analysis (D):* The explication of the life-history in which experiences and position-taking are embedded. The patient's life-history is the personal context within which her experiences may become meaningful. All of any person's life-events (including those that at face value look meaningless) are, according to psychodynamics, lawful and potentially meaningful in a particular way for that person. Also, all psychological events have at least as one of their motivations a psychological one and can thereby become meaningful on a psychological basis.

## Conclusions

Since the first decades of the twentieth century, the phenomenological branch of psychopathology has been developing more in-depth understandings of mental disorders. Recently, the frontiers of phenomenological psychopathology have expanded to the productive development of therapeutic strategies that target the whole of existence in their actions. The way we understand human existence determines how we understand psychopathological experiences and, especially, how we behave toward such patients. Phenomenology offers a radically human way of practicing psychiatry, a way that captures existence in all its determinations and singularities.

## Author contributions

GM wrote the manuscript and discussed suggestions from the co-authors. MT contributes to the conception of the work and co-wrote its manuscript. MM helped drafting the work and contributed to its conception. GS contributed to the conception of the manuscript and critically discussed its content.

### Conflict of interest statement

The authors declare that the research was conducted in the absence of any commercial or financial relationships that could be construed as a potential conflict of interest.
